# Impact of Chemical and Alternative Fungicides Applied to Grapevine cv Nebbiolo on Microbial Ecology and Chemical-Physical Grape Characteristics at Harvest

**DOI:** 10.3389/fpls.2020.00700

**Published:** 2020-05-29

**Authors:** Kalliopi Rantsiou, Simone Giacosa, Massimo Pugliese, Vasileios Englezos, Ilario Ferrocino, Susana Río Segade, Matteo Monchiero, Ivana Gribaudo, Giorgio Gambino, Maria Lodovica Gullino, Luca Rolle

**Affiliations:** ^1^Department of Agricultural, Forest and Food Sciences, University of Turin, Grugliasco, Italy; ^2^Agroinnova-Centre of Competence for the Innovation in the Agro-Environmental Sector, University of Turin, Grugliasco, Italy; ^3^ANT-NET srl, Turin, Italy; ^4^Institute for Sustainable Plant Protection, National Research Council (IPSP-CNR), Turin, Italy

**Keywords:** antifungal compounds, texture analysis, microbial ecology, grapevine berries, harvest, powdery and downy mildews

## Abstract

Viticulture is a cropping system in which treatment against fungal diseases (in particular powdery and downy mildews) can be extremely frequent. Accordingly, a reduction in antimicrobial treatments and the application of environmentally-friendly compounds are becoming increasingly important for a more sustainable viticulture. In addition to their effect against pathogens, the impact of these products on the quality of the grapes is very important for the oenological industries, but unfortunately at present few data are available. We evaluated the effect of the application of biocontrol products and resistance inducers in the vineyard on the mechanical properties, microbial ecology, technological and phenolic maturity of *Vitis vinifera* “Nebbiolo” grapes at harvest. The yield and vigor of vines were not influenced by the treatments, nor were the production of primary and secondary metabolites. However, the active ingredients influenced the mechanical properties of the skin (hardness and thickness). A significant hardening of the skin was detected when laminarin and chito-oligosaccharides were used, and sulfur induced a thickening of the skin with potential consequences for wine quality. Furthermore, the yeast community present on grape berries was influenced by the treatments. The abundance of *Aureobasidium pullulans*, the dominant species on the grape berry, changed in response to the compounds used. In addition, *Alternaria* sp. was reduced in some treatments with a potentially positive effect on the quality and the safety of the grapes. This study provides an overview of the effect of biocontrol products and resistance inducers on microbial ecology and “Nebbiolo” grape quality, contributing to the establishment of more sustainable and effective defense strategies in viticulture.

## Introduction

Powdery and downy mildews are among the key fungal diseases of European grape-growing regions, including Italy, significantly impacting crop quality and quantity. Powdery mildew is caused by *Erysiphe necator*, a polycyclic fungus infecting all green tissues with gray-white mycelia on their surface. *E. necator* can cause a reduction in grape yield of up to 45%, as a consequence of smaller diseased berries (Calonnec et al., 2004). In addition, severely infected clusters have higher total acidity than healthy ones, a higher concentration of phenylacetic acid, acetic acid and γ-decalactone, and a lower content of total anthocyanin and 3-mercaptohexanol (Calonnec et al., 2004; Pinar et al., 2017). Downy mildew is caused by the oomycete *Plasmopara viticola*, affecting the leaves and berries under warm, moist and humid environmental conditions. *P. viticola* may reduce grape yield and affects the aroma and flavors in wine, with organoleptic impact of the pathogen infection differing according to specific grape variety and impacts on berry quality and on the ability of a wine to age (Pons et al., 2018). These diseases are mainly controlled using chemical fungicides in conventional vineyards, where problems with pesticide residues and resistance to active ingredients are increasing. Copper and sulfur are used on organic farms (Gessler et al., 2011; Gadoury et al., 2012).

Viticulture is one of the most-frequently treated cropping systems (Komarek et al., 2010; Gill and Garg, 2014; Tsakirakis et al., 2014; Provost and Pedneault, 2016). Consequently, restrictions have been applied by the European Commission on the number of pesticide treatments (Directive 2009/128/EC) and on the maximum quantity per year of copper fungicides (Regulation 2002/473/EC). Recently, copper, which is still considered the most important antifungal product in organic viticulture, has been added to the list of candidates for substitution (European Commission Implementing Regulation 2018/84) and further limited to 4 kg per hectare/year spread over 7 years from February 1st 2019 (European Commission Implementing Regulation 2018/1981 of 13 December 2018). In parallel, there is increasing demand by consumers for organic and more environmentally-friendly products and this trend is affecting the market of the viticulture and wine sectors (Schäufele and Hamm, 2017).

This situation has encouraged the development of alternative crop protection strategies and of the use of more environmentally-friendly products, such as biofungicides, resistance inducers and biostimulants against powdery and downy mildews (Gadoury et al., 2012; Thind, 2017; Gutiérrez-Gamboa et al., 2019), with a view to achieving sustainable production (Flores, 2018). As a result, an increasing number of farmers have begun to apply only biocontrol products to grapevines, considering that they can be applied up to a few days before harvest and during full bloom. Biocontrol products are plant protection products containing micro-organisms (fungi, bacteria, viruses, etc.) or natural substances of mineral, plant or microbial origin, with a lower environmental impact compared to synthetic chemical products. Examples of these products are laminarin, chitosan and bicarbonate. In particular, laminarin is extracted from the brown algae *Laminaria digitata*, is known to induce resistance against downy and powdery mildew and gray mold in grapes (Aziz et al., 2003; Gauthier et al., 2014; Chalal et al., 2015; Romanazzi et al., 2016; Pugliese et al., 2018). According to Trouvelot et al. (2014), chitosan has been studied as plant protectant for more than 30 years and chitosan and chito-oligosaccharides have also been applied to control downy mildew on detached leaves (Aziz et al., 2006; Trotel-Aziz et al., 2006), with a variable control efficacy, and a decrease in the concentration of several amino acids has been observed on treated grapes (Garde-Cerdàn et al., 2017). Salts such as bicarbonates have been used successfully for the control of powdery and downy mildews (Deliopoulos et al., 2010; Lukas et al., 2016).

Due to a lack of information in the scientific literature, agronomists and wine growers are often not aware of the impact that these new products can have indirectly on the quality of grapes. In particular, few data are available on their influence and/or interaction in the synthesis of primary and secondary metabolites, on the composition of the skin cell wall and related mechanical properties, and on the microbiota present on the grape clusters.

The above-mentioned induced resistance can represent an attractive strategy for increasing the production of plant defense compounds in grapevine, such as phenolic compounds (Flamini et al., 2013). For red wine production, especially, the content and chemical characteristic of phenolic compounds in berry skins and seeds at harvest play an essential role in the sensory properties of the resulting product, impacting on the chromatic characteristics, astringency and bitterness (Preys et al., 2006; Paissoni et al., 2018). Moreover, the final phenolic composition of wine is related to the capacity of the grape skin and seeds to release these compounds during the maceration-fermentation process. Regarding berry skins, this phenomenon, conventionally called “extractability,” may be influenced by phytosanitary treatments affecting physical-mechanical characteristics (i.e., hardness and thickness) (Rolle et al., 2012a).

The grape surface is colonized by different microorganisms, mainly filamentous fungi, bacteria and yeasts, which in relation to the final product may have positive or negative effects (Milanović et al., 2013; Alessandria et al., 2015). Several environmental factors, such as geographical location, climatic conditions (temperature, rainfall, wind) and vineyard treatments, affect the microbial communities present on the grape surface at harvest (Barata et al., 2012; Bokulich et al., 2014; Zhang et al., 2019). Many of those factors may also interact, impacting on the microbial diversity and, consequently, wine quality (Barata et al., 2012). The importance of the grape microbiota, especially in the first stages of the spontaneous or inoculated alcoholic fermentation, is well documented (Cravero et al., 2016). In particular, non-inoculated fermentation, a practice that winemakers regard as enhancing regional typicity, is also popularly considered to enhance wine quality through the production of a wide spectrum of secondary compounds (Ciani et al., 2010). Considering the importance of the indigenous must microbiota on wine composition, there is an increasing interest in understanding the effect of vineyard plant protection products on species distribution and persistence on the grapes at harvest. To this end, studies have investigated the impact of organic and conventional agrochemicals on grape microbiota in a wide range of grape varieties (Schmid et al., 2011; Piao et al., 2015; Agarbati et al., 2019). Despite these extensive researches, little is known about the impact of a wide spectrum of biocontrol products and of resistance inducers on microbial diversity at harvest.

In the present paper, alternative strategies against downy and powdery mildews, based on the application of biocontrol products and resistance inducers, were applied in a vineyard of “Nebbiolo,” a wine grape variety commonly used in the production of renowned Barolo, Barbaresco and Roero wines. Our main purpose was to evaluate the effect of these products on (i) the parameters involved in the technological and phenolic maturity of the grapes at harvest, and (ii) the microbial ecology, as determined by culture-dependent and culture-independent approaches.

## Materials and Methods

### Field Trial

The field trial was conducted on wine grape “Nebbiolo” (*Vitis vinifera* L.) in a vineyard located in Piobesi D’Alba (North-West Italy) (GPS: 44.731760, 7.988324, hill area) during 2017. Vines were planted in parallel and contiguous rows, vertically trained, Guyot pruned and all grafted onto the same rootstock (Kober 5BB). The distance between vines was 0.90 × 2.5 m. Vineyard management during the growing season was uniform in the experimental site and in compliance with regional best agricultural practices. The experimental scheme included four randomized blocks per treatment, each containing eight plants along a row, and the treatments were assigned to plots using a random-number generator. Best agricultural practices were considered in order to avoid drift of product from one plot to another, such as avoiding carrying out the treatments if wind speed was higher than 2 km/h and the direction was perpendicular to the rows. The experimental products being tested were applied with a hand-pulled 2-stroke engine sprayer at a pressure of 15 bar, distributing 400–600 l/ha. The plants were treated in accordance to the guidelines EPPO/OEPP PP 1/31 (3) (EPPO, 2019).

The commercial antifungal products were used according to the manufacturer’s instructions. The products were compared with a non-treated control (CTR), a standard control strategy (S+Met) consisting of sulfur and metiram (until the end of flowering) and sulfur and copper hydroxide (until the end of the season) applied in conventional farms in the region, and a standard control strategy (S) applied in organic farms in the region.

The commercial formulations applied were: acibenzolar-S-methyl (50% a.i., Bion, Syngenta Crop Protection); fosetyl-Al (80% a.i., Aliette, Bayer Crop Science); potassium phosphonate (755 g/l a.i., Century, BASF Agro); laminarin (Vacciplant 45 g/l a.i., Arysta Lifescience); chito-oligosaccharides and oligogalacturonides (12.5 g/l a.i., Ibisco, Gowan); potassium bicarbonate (85% a.i., Armicarb, SCAM); sulfur (80% a.i., Thiovit Jet, Syngenta Crop Protection); calcium oxide (22.1% a.i., Califol, AgriNewTech srl); metiram (70% a.i., Polyram, BASF Agro); copper hydroxide (20% a.i., Coprantol Hi Bio 2.0, Syngenta Crop Protection); electrolyzed water (sodium hypochlorite, EVA System^®^, De Nora S.p.A.). These commercial formulations were applied in combination with sulfur and copper, as shown in Table 1. The efficacy of the different treatments was evaluated prior to harvest analyzing the percentage of affected grape clusters and the percentage of affected berries by powdery and downy mildew, i.e., incidence and severity, as described in Pugliese et al. (2018) and according to the guidelines EPPO/OEPP PP 1/31 (3) (EPPO, 2019). In order to avoid that the drift of product from one plot to another along the same row may influence the results, only the clusters of 4 plants out of 8 and located at the center of each plot were considered for the evaluation. Analysis of variance was carried out with the statistical program SPSS 22.0 and, after ANOVA, Tukey’s “HSD” was used as *post hoc* analysis with a significance defined at the *p* < 0.05 level.

**TABLE 1:** List of tested treatments, active ingredients, dosages, period and number of applications.

Treatment	Active ingredient (a.i.)	Dose a.i. (g/ha)	Days between applications	Period of application*	Number of applications
CTR	Non-treated control	–	–	–	–
AcS-Mt	Acibenzolar-S-methyl	100	7–9	Till bunch closure	6
	Sulfur + Copper hydroxide	3200+600	7–9	From bunch closure to harvest	9
Fos-Al	Fosetyl-Al	2000	7–9	Till bunch closure	6
	Sulfur + Copper hydroxide	3200+600	7–9	From bunch closure to harvest	9
K-Pho	Potassium phosphonate	3020	7–9	Till bunch closure	6
	Sulfur + Copper hydroxide	3200+600	7–9	From bunch closure to harvest	9
Lam	Laminarin +Metiram	90 + 1400	7–9	Till fruit set	7
	Laminarin + Copper hydroxide	90 + 600	7–9	From fruit set to harvest	8
Chito	Chito-oligosaccharides and oligogalacturonides +Metiram	2500+1400	7–9	Till fruit set	7
	Chito-oligosaccharides and oligogalacturonides + Copper hydroxide	2500+600	7–9	From fruit set to harvest	8
K-Bic	Potassium bicarbonate+Metiram	4250+1400	7–9	Till fruit set	7
	Potassium bicarbonate+ Copper hydroxide	4250+600	7–9	From fruit set to harvest	8
S+Met	Sulfur + Metiram	3200+1400	7–9	Till fruit set	7
	Sulfur + Copper hydroxide	3200+600	7–9	From fruit set to harvest	8
CaO	Calcium oxide	884	7–9	All growing season	15
EOW	Electrolyzed water	10% solution	7–9	All growing season	15
S	Sulfur	3200	7–9	All growing season	15
Met	Metiram	1400	7–9	Till fruit set	7
	Copper hydroxide	600	7–9	From fruit set to harvest	8

S+Met, standard control strategy applied in conventional farms; S, standard control strategy applied in organic farms in the region. *Applications started on April 24th 2017 and ended on August 25th 2017. Bunch closure on June 7th 2017, fruit set on June 16th 2017.

### Agronomic Parameters and Grape Sampling

At harvest, total fruit yield (kg) and number of clusters were determined for each treatment and block. Mean cluster weight was calculated. Plant vigor was estimated in winter as pruned wood weight.

Berry sampling was performed in the vineyard at harvest. For each treatment, 200 grape berries (50 per block) with pedicel attached were randomly selected and aseptically collected into sterile sampling bags. The samples were transported in the laboratory under refrigerated conditions and analyzed immediately.

### Chemical Analysis of Grape Must

For each treatment-block combination, 50 g of intact berries were crushed, and the liquid grape must was obtained. After centrifugation (3000 × *g*, 20 °C, 15 min), the supernatant was used for the determination of the following technological parameters: soluble solids content, as °Brix, using an Atago Palette 0–32 (Atago, Tokyo, Japan) pH, by potentiometry (InoLab 730, WTW, Weilheim, Germany); and total acidity, following the OIV-MA-AS313-01 method (OIV, 2017).

### Berry Skin Phenolic Composition

The evaluation of the berry skin phenolic composition (i.e., anthocyanin, oligomeric and polymeric flavonol contents) was conducted for each treatment and block, following the Di Stefano and Cravero (1991) method with Río Segade et al. (2014) modifications. Ten berries for each sample were weighed and peeled, and the skins obtained were quickly placed in 40 mL of a hydroalcoholic buffer solution prepared as follows: 12% (v/v) ethanol, 5 g/L tartaric acid, 2 g/L sodium metabisulfite, and afterward the pH was adjusted to pH 3.2. Then, the skins were macerated for 24 h, and afterward homogenized using an Ultra-Turrax T25 homogenizer (IKA, Staufen, Germany) set at 8000 rpm for 1 min. After centrifugation (3000 × *g*, 20°C, 15 min), the supernatant was taken and brought to 50 mL with the above-mentioned buffer solution and used as grape skin extract in subsequent analysis.

The phenolic indices were determined by spectrophotometry, using a Shimadzu UV-1800 machine (Shimadzu Corporation, Kyoto, Japan). The total anthocyanins and flavonoids content was determined after dilution of the grape skin extract using a 70:30:1 (v/v) ethanol:water:hydrochloric acid (concentrated) solution. Proanthocyanidins assay required acid-catalysis of the polymerized flavanols under heat. Vanillin assay was performed by binding the vanillin reagent to the free 6 and 8 positions of monomeric-oligomeric flavanols in acid medium.

### Grape Texture Analysis

Ten intact berries were randomly taken at harvest from each treatment and block, thus resulting in 40 berries for each treatment, and used for the determination of their instrumental mechanical properties. The skin parameters detected were berry skin break force (F_sk_) and berry skin thickness (Sp_sk_), following the method proposed by Letaief et al. (2008). Briefly, for the first test each intact berry was placed alone on the platform of a TA.XTplus Texture Analyser (Stable Micro Systems, Godalming, Surrey, United Kingdom) and was subjected to penetration by a standardized stainless steel needle (P/2N probe, Stable Micro System) at a constant speed of 1 mm/s. The maximum resistance of the berry skin to the needle penetration is defined as the berry’s F_sk_, expressed in N. For the second test, Sp_sk_, a portion of the skin was obtained from each berry by peeling and removing pulp residues. The berry skin portion was placed on the flat platform of the above-mentioned instrument, and a probe composed of a flat 2 mm stainless steel cylinder (P/2 probe, Stable Micro Systems) began the descent at 0.2 mm/s toward the sample. In this way, the machine acted as a precise caliper and was able to detect the thickness of the berry portion (Sp_sk_), expressed in μm.

### Microbiological Analysis of Grapes

For each treatment-block combination, 50 g of single grape berries were placed in sterile bottles containing 450 mL of a solution of 0.1% peptone and 0.001% Tween 80 (Sigma, Milan, Italy). The bottles were stirred for 30 min at 150 rpm to facilitate the release of cells from the berry surface of the grapes in the solution. The rinse solution was serially diluted in peptone water (0.1% peptone). Aliquots of the dilutions were plated as follows. Wallerstein Laboratory Nutrient Medium agar (WLN, Biogenetics, Milan, Italy) was used for enumeration of fungi. The medium was spread plated and incubated at 30°C for 5 days. MRS agar (Biogenetics) supplemented with Delvocid (25 μg/mL, DSM Specialties, Heerlen, Netherlands) was used for enumeration of lactic acid bacteria and was pour plated and incubated at 30°C for 1 week. GYC [10 g/L yeast extract (Biogenetics), 20 g/L CaCO_3_ (Sigma), 20 g/L agar, 20 mL/L ethanol (99% v/v, Sigma)] was used for enumeration of acetic acid bacteria and spread plated and incubated at 30°C for 1 week. After incubation, the colonies were counted and mean and standard deviation values were calculated. Yeast colonies formed on WLN plates were counted on the basis of the color and morphology as described previously by Urso et al. (2008). Ten yeast isolates from each colony morphotype were picked, purified by streaking on WLN medium and subjected to molecular identification to species level.

### Molecular Identification of Yeast Isolates

Isolates randomly selected from the WLN plates were purified by streaking on WLN and then grown overnight in 1 mL YPD medium (2% w/v yeast extract, 1% w/v peptone, 2% w/v dextrose, all from Biogenetics) at 30°C. The broth culture was centrifuged at 14,000 × *g* for 5 min and the pellet was subjected to DNA extraction according to Cocolin et al. (2000). The extracted DNA was quantified using a Nanodrop ND-1000 spectrophotometer (Celbio, Milan, Italy) and standardized at 100 ng/μL. The ITS1-5.8S rRNA-ITS2 (ITS) region was PCR amplified and subjected to restriction fragment length polymorphism (RFLP) for all isolates (Esteve-Zarzoso et al., 1999). The ITS region was amplified with primers ITS1 (5′- TCCGTAGGTGAACCTGCGG -3′) and ITS4 (5′-TCCTCCGCTTATTGATATGC 3′) (White et al., 1990). The reaction mix was 50 μL and contained 10 mM Tris–HCl (pH 8.3), 50 mM KCl, 1.5 mM MgCl_2_, 0.2 mM of deoxynucleoside triphosphates (dNTPs), 1.25 U of Taq Polymerase (Applied Biosystems, Milan, Italy), 0.2 μM of each primer and 100 ng of template DNA. Amplification was carried out using a PTC-200 DNA Engine MJ Research Thermal Cycler (Bio-Rad, Milan, Italy), as described by Esteve-Zarzoso et al. (1999), and the PCR products were checked by electrophoresis on 1.5% (w/v) agarose gel. The PCR products were subsequently digested with endonucleases *HinfI*, *HaeIII*, *CfoI* (Promega, Milan, Italy), according to the supplier’s instructions. The restriction fragments were separated by electrophoresis in 3% agarose gel and stained with ethidium bromide. PCR and RFLP fragment lengths were used to identify yeasts by comparing the restriction bands with those available in the literature (Guillamon et al., 1998; Esteve-Zarzoso et al., 1999; Granchi et al., 1999; Sabate et al., 2002). Identification to species level was confirmed by sequencing the D1-D2 loop of the 26S rRNA encoding gene, after amplification using primers NL1/NL4 (Kurtzman and Robnett, 1997) to obtain a PCR product, which was sequenced by a commercial facility (Eurofins, Germany).

### Mycobiota Amplicon Target Sequencing

Ten mL of the grape rinse was centrifuged for 10 min at 14,000 × *g* and the supernatant was discarded. The pellet was used for total DNA extraction using the Master Pure Complete DNA and RNA Purification Kit (Illumina Inc, San Diego, CA, United States) following the manufacturer’s instructions. DNA was quantified using the Qubit dsDNA assay kit (Thermo Fisher Scientific) and standardized at 5 ng/μL. DNA was used to assess the mycobiota by targeting the D1-D2 domain of the 26S rRNA gene using a modification of the primer LS2F (5′-GAG TCG AGT TGT TTG GGA AT-3′) and NL4R (5′-GGT CCG TGT TTC AAG ACG G-3′), as recently described (Mota-Gutierrez et al., 2019).

PCR mixtures (25 μL) were prepared using 12.5 μL of the 2X Kapa HiFi HotStart ReadyMix Taq (Roche, Milan, Italy), 1 μM of each primer, 2.5 μL of DNA template, and PCR-grade water. Thirty cycles of 30 s of denaturation (95°C), 30 s of primer annealing (55°C), and 30 s of primer elongation (72°C), followed by a final elongation step (72°C) of 10 min, were performed. The amplicons’ size and quality were assessed using a Bio-Rad Experion Workstation (Bio-Rad, Milan, Italy). In a second PCR step, amplicons were combined with the sequencing adapters and dual indices using the Nextera XT Index Kit (Illumina, San Diego, CA, United States), forming the multiplexed paired-end libraries, and quantified using the Qubit dsDNA Assay Kit (Thermo Fisher Scientific, Milan, Italy). Individual libraries were diluted to 4 nM, denaturated with 0.2 N NaOH and spiked with 30% (v/v) PhiX. The combined pool library and PhiX were diluted to 12 pM and paired-end sequencing was performed on a MiSeq platform, using MiSeq Reagent Kit v3 2 × 250 cycle (Illumina, San Diego, CA, United States), following the standard Illumina sequencing protocol. The software used for the base calling and Illumina barcode demultiplexing processes were the Miseq Control Software version. The 26S raw read data were deposited in the Sequence Read Archive of NCBI under BioProjectID PRJNA575160.

### Wine Production and Analysis

Experimental wines were produced from three treatments: CTR, potassium bicarbonate (K-Bic), and S+Met. A standard vinification protocol was applied for each treatment considered and two replicates of 100 kg of grapes were processed in the experimental cellar of University of Turin under controlled conditions. In brief (Boccacci et al., 2020): grapes harvested were crushed in a TEMA destemmer–crusher (Enoveneta, Piazzola sul Brenta, Italy). The mash was added with 25 mg/L of potassium metabisulfite. After about 6 h, selected *Saccharomyces cerevisiae* yeasts (Lalvin BRL97, Lallemand, Inc., Montreal, Canada) were inoculated at a dose of 20 g/hL. Two punch-downs per day were carried out in the first 3 days, then two pumping-overs per day until the end of maceration, which lasted 10 days. The end of macerations was followed by the gentle pressing of the pomace cap using a PMA 4 pneumatic press (Velo SpA, Altivole, Italy) with a maximum pressure of 1.2 bar, and a small aliquot of the press wine was joined to the free-run wine. Wine was inoculated with *Oenococcus oeni* Lalvin VP41 strain (Lallemand) to induce malolactic fermentation (MLF). Once MLF was completed, the wines were racked to remove lees, and free SO_2_ concentration was adjusted to 70 mg/L. The alcoholic fermentation (AF) and MLF were carried out at controlled temperatures of 27 ± 2 and 20 ± 1°C, respectively. Wines were cold-stabilized at 0°C for 2 weeks, filtered (Seitz K300 grade filter sheets, Pall Corporation, Port Washington, NY, United States) and then bottled in glass bottles of 0.75 L with cork stoppers.

After 5 months of bottle storage, wines were analyzed. Following parameters were determined: ethanol content, residual sugars, glycerol, and organic acids by HPLC using the chromatographic condition proposed by Giordano et al. (2009), while pH and titratable acidity were evaluated using OIV (2017) methods. Wine phenolic composition was determined through spectrophotometric methods using an UV-1800 spectrophotometer (Shimazdu Corp., Kyoto, Japan): total polyphenolic index was assessed by Folin-Ciocalteu reagent assay, proanthocyanidins determined after acid hydrolysis at 100°C using a ferrous salt (FeSO_4_) as catalyst according to the Bate-Smith reaction and expressed as mg of cyanidin chloride/L of wine, monomeric and oligomeric forms of flavanols were evaluated as flavanols reactive to vanillin and expressed as mg of (+)-catechin/L of wine (Petrozziello et al., 2018). Total anthocyanins were determined by measuring absorbance at 536–540 nm after dilution with a hydroalcoholic solution composed of ethanol: water: 37% hydrochloric acid (70:30:1, v/v) and expressed as mg of malvidin-3-glucoside chloride/L of wine (Petrozziello et al., 2018). After the acquisition of visible spectra of undiluted samples using 1-mm optical path cuvettes, color intensity was calculated as the sum of absorbance measured at 420, 520, and 620 nm (A420 + A520+ A620 on an optical path of 10 mm) and hue was obtained as the ratio of absorbances measured at 420 and 520 nm (A420/A520) following the method OIV-MA-AS2-07B (OIV, 2017). The wine color was also evaluated by the CIE L^∗^a^∗^b^∗^ parameters, namely lightness (L^∗^), red/green color coordinate (a^∗^), and yellow/blue color coordinate (b^∗^), according to the method OIV-MA-AS2-11. The total color difference (ΔE^∗^) between two samples (for example between a fined wine and the respective control wine) was calculated using the following expression: ΔE^∗^ = [(ΔL^∗^)^2^ + (Δa^∗^)^2^ + (Δb^∗^)^2^]^1/2^ (OIV, 2017).

### Bioinformatics and Statistical Analysis

Paired-end reads were first merged using FLASH software (Magoc and Salzberg, 2011), with default parameters. Joined reads were quality trimmed (Phred score < 20) using QIIME 1.9.0 software (Caporaso et al., 2010) and short reads (<300 bp) were discarded using Prinseq (Schmieder and Edwards, 2011). OTUs were clustered at 99% similarity by means of UCLUST clustering methods (Edgar, 2010) and representative sequences of each cluster were used to assign taxonomy to a reference 26S database by means of the RDPII classifier (Wang et al., 2007). An *ad hoc* database (Mota-Gutierrez et al., 2019) was created with the reference 26S gene sequences and the taxonomy. This database was used in the QIIME pipeline to obtain the OTU table and the downstream output.

Sequences were double-checked using the BlastN search tool^1^ to confirm the taxonomy assignment. In order to avoid biases due to different sequencing depths, all samples were rarefied at 16 564 reads after raw read quality filtering, and the OTU table was filtered for OTUs occurring at 0.2% of the relative abundance in at least two samples. Statistics and plotting were carried out in the R environment^2^. Alpha diversity indexes (Shannon, chao1 and n° of observed species) were calculated by the function *vegan* in R (Dixon, 2003) and was further analyzed using the Pairwise Kruskal-Wallis test in order to find significant differences in microbial taxon abundance according to treatment. The OTU table was used to perform the Adonis statistical test with 999 permutations through the vegan package of R. The OTU table displays the highest taxonomy resolution that was reached; when the taxonomy assignment was not able to reach species level, the genus or family name was displayed. The OTU table was used to build a principal-component analysis (PCA) as a function of treatment by using the made4 package of R. Kruskal-Wallis tests were used to find significant differences in microbial taxa abundance according to treatment. Pairwise Spearman’s correlations were used to study the relationships between the relative abundance of mycobiota with disease index as well as with the berry skin mechanical properties. The correlation plots were visualized in R using the *corrplot* package of R. *P* values were adjusted for multiple testing using the Benjamini-Hochberg Procedure, which assesses the false discovery rate (FDR).

To test for significant differences in agronomic parameters, berry mechanical properties and must chemical compositions, the analysis of variance (ANOVA) was used (SAS statistical software, version 8.2, SAS Institute, Cary, NC, United States). Significant differences were highlighted at *p* < 0.05.

## Results and Discussion

### Disease Incidence

**FIGURE 1:**
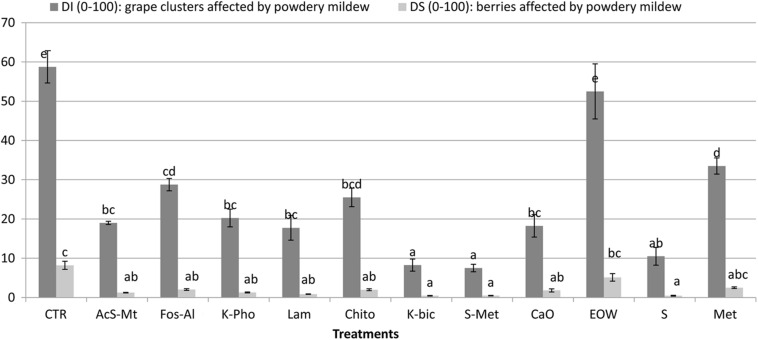
Efficacy of the treatments against *Erysiphe necator* on the incidence and severity on grape clusters, compared to non-treated control (CTR) and standard treatments for conventional (S+Met) and organic (S) farms in the region. Different letters mean significant differences (*p* < 0.05). Treatment descriptions are reported in Table 1.

The “Nebbiolo” vineyard used in the study was not infected with *P. viticola* and no symptoms caused by downy mildew were observed. Conversely, 58% of clusters were affected by *E. necator*, with a severity of 8% on non-treated control (CTR). Comprehensive details of the phytopathological results of this trial are reported in Figure 1. Potassium bicarbonate (K-Bic) was the most efficient in reducing the disease, similar to the standard treatments (S+Met and S). In addition, treatments using acibenzolar-S-methyl (AcS-Met), potassium phosphonate (K-Pho), laminarin (Lam), chito-oligosaccharides and oligogalacturonides (Chito) and calcium (CaO) were also effective compared to the standard treatment with sulphur (S). Fosetyl-Al (Fos-Al) and metiram (Met) had a lower efficacy and similar to Chito. Electrolyzed water (EOW) was not effective.

### Agronomic Parameters and Chemical Analysis of Grape Must

The data on yield components collected at harvest, as well as the vigor estimation at the end of the vegetative season, did not produce significant differences related to the various treatments applied (Table 2). The incidence of fungal pathogens on leaves and berries reported above did not seem to have substantial effects, compared with the CTR, on the quantitative performance of the vines in the particular season in which the trial was conducted.

**TABLE 2:** Field performances of “Nebbiolo” vines subjected to different treatments.

Treatment	Yield (kg/plant)	No. clusters/plant	Ave. cluster weight (g)	Pruning wood (kg/plant)
CTR	2.18 ± 0.39	9.7 ± 1.5	226.2 ± 29.3	0.60 ± 0.34
AcS-Mt	2.55 ± 0.58	10.9 ± 1.0	231.2 ± 34.1	0.62 ± 0.42
Fos-Al	2.35 ± 0.82	10.4 ± 2.3	220.7 ± 37.1	0.73 ± 0.32
K-Pho	2.24 ± 1.10	8.9 ± 2.3	241.5 ± 53.4	0.68 ± 0.37
Lam	1.95 ± 0.56	9.0 ± 2.6	215.7 ± 12.3	0.61 ± 0.25
Chito	2.00 ± 0.20	7.7 ± 2.4	291.2 ± 131.6	0.76 ± 0.42
K-Bic	2.14 ± 0.56	9.5 ± 1.3	223.5 ± 33.2	0.65 ± 0.30
S+Met	3.46 ± 1.38	12.8 ± 3.3	263.2 ± 43.4	0.78 ± 0.40
CaO	2.91 ± 0.72	12.4 ± 1.5	234.7 ± 45.1	0.79 ± 0.32
EOW	2.13 ± 0.27	9.5 ± 1.0	226.2 ± 45.3	0.60 ± 0.42
S	2.35 ± 0.73	10.5 ± 1.4	223.5 ± 62.7	0.70 ± 0.29
Met	2.69 ± 0.75	12.3 ± 3.2	219.5 ± 31.9	0.71 ± 0.41
Sign.	0.215	0.056	0.781	0.999

*Data expressed as average ± standard deviation (*n* = 4). Sign.: *p*-values are shown for each column. Treatment descriptions are reported in Table 1.*

In addition, the chemical parameters, which define the so-called technological maturity of the grapes, did not show significant differences among the different treatments (Table 3). Sugar content, pH and total acidity presented homogeneous values among the different treatments, with data in agreement with previous studies (Cagnasso et al., 2011). Therefore, for the primary metabolites also, the treatments used to control downy and powdery mildews did not have a substantial interference or elicitor effect. Other studies have also highlighted that grape musts from Montepulciano grapevines treated with laminarin and copper hydroxide were not significantly different from control in terms of sugar content, pH and titratable acidity (Romanazzi et al., 2016).

**TABLE 3:** Must chemical parameters and berry skin mechanical properties of “Nebbiolo” grapes harvested from each treatment.

Treatment	°Brix	pH	Total acidity (g/L as tartaric acid)	Berry skin break force (F_sk_, N)	Berry skin thickness (Sp_sk_, μm)
CTR	24.70 ± 0.34	3.26 ± 0.01	6.07 ± 0.39	0.603 ± 0.101^ab^	176 ± 25^a^
AcS-Mt	24.40 ± 0.47	3.28 ± 0.03	6.17 ± 0.23	0.608 ± 0.106^ab^	186 ± 27^ab^
Fos-Al	23.93 ± 0.36	3.30 ± 0.04	5.74 ± 0.39	0.574 ± 0.099^ab^	198 ± 21^bc^
K-Pho	24.40 ± 0.55	3.27 ± 0.05	5.69 ± 0.38	0.607 ± 0.133^ab^	191 ± 30^abc^
Lam	24.70 ± 0.28	3.25 ± 0.05	5.81 ± 0.22	0.627 ± 0.103^b^	195 ± 27^abc^
Chito	24.28 ± 1.02	3.27 ± 0.05	5.86 ± 0.61	0.629 ± 0.103^b^	194 ± 34^abc^
K-Bic	24.13 ± 0.49	3.28 ± 0.05	5.70 ± 0.42	0.547 ± 0.110^a^	198 ± 31^bc^
S+Met	23.90 ± 1.04	3.28 ± 0.02	5.77 ± 0.43	0.563 ± 0.085^ab^	198 ± 28^bc^
CaO	24.48 ± 0.40	3.21 ± 0.04	5.97 ± 0.71	0.599 ± 0.115^ab^	188 ± 23^ab^
EOW	24.23 ± 1.04	3.25 ± 0.02	5.90 ± 0.39	0.587 ± 0.124^ab^	199 ± 22^bc^
S	24.23 ± 0.55	3.27 ± 0.07	5.67 ± 0.64	0.594 ± 0.121^ab^	209 ± 28^c^
Met	23.95 ± 1.38	3.23 ± 0.04	5.93 ± 0.59	0.567 ± 0.101^ab^	191 ± 25^abc^
*Sign.*	0.891	0.119	0.761	0.014	<0.001

*Data expressed as average ± standard deviation (*n* = 4 for grape must parameters, *n* = 40 for berry skin mechanical parameters). Different letters inside each column means significant differences at *p* < 0.05 (Tukey-*b* test). Treatment descriptions are reported in Table 1.*

### Phenolic Composition of Berry Skin

The effects of the treatments on the grape skin phenolic composition are reported in Table 4, using the main spectrophotometric indexes used by operators of the viticulture-oenology sector. Due to high variability of data among replicates, no statistical differences in anthocyanin, polymeric flavanol (proanthocyanidins) and oligomeric flavanol (reactive to vanillin) contents were found among the different treatments. However, low average values of total anthocyanin content were detected using Calcium oxide (CaO), while high total flavanols content (polymeric + oligomeric) was detected after the application of chito-oligosaccharides and oligogalacturonides (Chito). Despite these small differences, it is possible to hypothesize that in “Nebbiolo” there is no real impact from the different treatments in the biosynthetic pathways of these secondary metabolites, as well as no elicitor effect.

**TABLE 4:** Berry skin phenolic content in “Nebbiolo” grapes harvested from each treatment.

Treatment	Total anthocyanin index (mg malvidin-3-glucoside chloride/g berry skins)	Total flavonoids(mg (+)-catechin/g berry skins)	Proanthocyanidins assay(mg cyanidin chloride/g berry skins)	Vanillin assay (mg (+)-catechin/g berry skins)
CTR	4.43 ± 0.34	35.34 ± 1.43	30.60 ± 2.05	8.57 ± 0.76
AcS-Mt	4.39 ± 0.54	35.85 ± 4.82	27.56 ± 4.67	8.14 ± 1.71
Fos-Al	4.90 ± 1.13	35.49 ± 8.99	30.65 ± 6.30	7.86 ± 2.11
K-Pho	4.27 ± 0.44	33.59 ± 4.30	30.86 ± 4.20	8.71 ± 1.76
Lam	4.33 ± 0.21	32.24 ± 1.18	32.40 ± 4.28	8.61 ± 0.82
Chito	4.37 ± 0.36	34.25 ± 1.50	33.54 ± 2.34	9.30 ± 0.95
K-Bic	4.80 ± 0.58	36.06 ± 3.86	30.73 ± 3.37	8.79 ± 1.95
S+Met	4.48 ± 1.11	32.81 ± 5.28	29.00 ± 1.98	8.02 ± 0.50
CaO	3.53 ± 0.23	29.03 ± 3.05	28.27 ± 3.40	8.35 ± 1.52
EOW	4.65 ± 1.30	33.58 ± 7.41	30.71 ± 4.79	9.39 ± 1.87
S	4.64 ± 0.78	33.24 ± 2.13	30.24 ± 4.11	7.68 ± 0.48
Met	4.26 ± 0.89	32.81 ± 7.55	28.63 ± 2.69	7.42 ± 0.37
*Sign.*	0.867	0.992	0.570	0.758

*Data expressed as average ± standard deviation (*n* = 4). Sign.: *p*-values are shown for each column. Treatment descriptions are reported in Table 1.*

### Mechanical Properties of Grapes

The effect of treatments on berry skin hardness, evaluated by skin break force parameter (F_sk_), and skin thickness (Sp_sk_) are reported in Table 3. Significant differences for F_sk_ were not found for any treatment when compared to CTR. Lam and Chito showed the significant highest values of F_sk_ (0.627 and 0.629 N, respectively) only with respect to K-Bic, whose grapes are characterized by lowest values of F_sk_ (0.547 N). K-Bic was one the best treatments against powdery mildew (Figure 1) with efficiency similar to standard chemical strategy S+Met, and more effective than Lam and Chito. The F_sk_ values found agree with data previously published for this variety (Rolle et al., 2012b). From a technological point of view, higher F_sk_ produces an increase in anthocyanin extraction yield and slower extraction kinetics during simulated maceration of “Nebbiolo” grape skin in wine-like solution (Rolle et al., 2008). The last aspect is particularly important for wine grape varieties rich in 3′-hydroxylated anthocyanins, such as “Nebbiolo,” because these pigments are extracted preferentially during the initial phase of maceration and may be easily oxidized by the enzymes present in the juice (González-Neves et al., 2008).

Despite the high standard deviation of data within each treatment, significant differences were found in berry Sp_sk_ with respect to CTR. The values of the Sp_sk_ parameter ranged from 176 to 209 μm for treatments CTR and S, respectively. Although all treatments impacted on the value of this mechanical property when compared to data for CTR berries, only after the application of Fos-Al, EOW, K-Bic, S+Met, and S a significant increase in skin thickness was observed (+22 –33 μm). Interestingly, the three most effective treatments against powdery mildew (S, S+Met and K-Bic) have shown higher values of skin thickness and this could suggest in “Nebbiolo” a correlation between this parameter and the resistance to *E. nector*. However, the same higher Sp_sk_ values were detected in EOW, the worst treatment for the control of powdery mildew without significant differences to the CTR, therefore further studies are needed. In general, even small variations in thickness can cause differences in terms of the extractability of phenolic substances, as reported by Río Segade et al. (2011) on “Galician” grapes. However, for “Nebbiolo” grapes treated with a specific inactive LSA extract in the vineyard, the berry skin thickening observed did not have a detrimental impact on anthocyanin extractability (Giacosa et al., 2019). Furthermore, skin thickness and hardness are factors that contribute to berries’ response to climatic adversities, regulate on-vine and off-vine withering kinetics (Rolle et al., 2011) and limit damage during harvest, packing, transport and storage (Kök and Çelik, 2004).

### Microbiological Quality of Grapes

**FIGURE 2:**
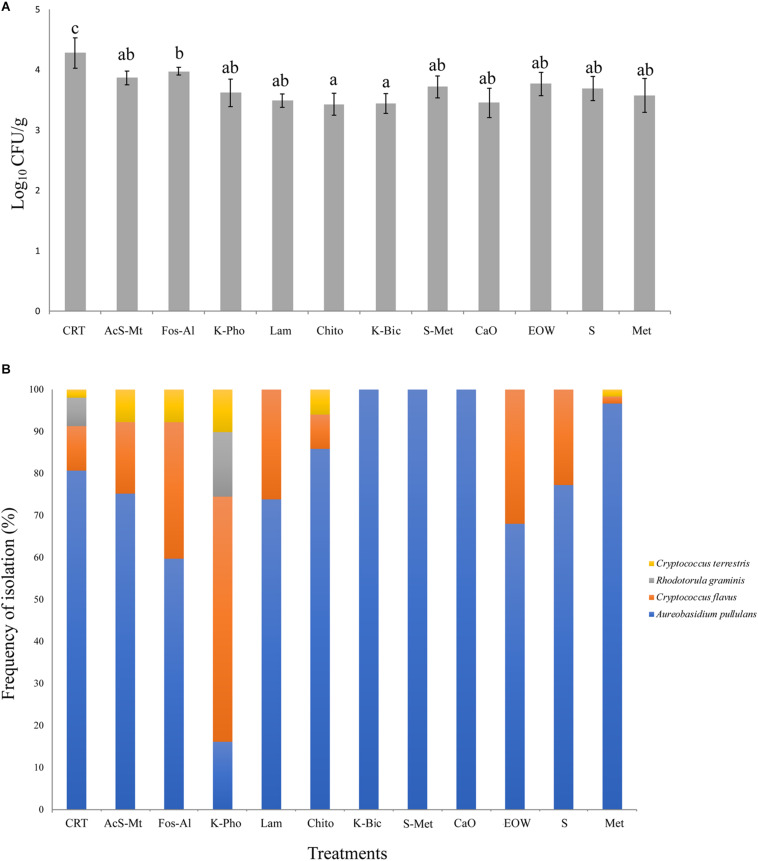
Fungal load, as determined by microbiological analysis on WLN medium, expressed in Log_10_ CFU/mL. The bars represent mean values and standard deviation from four replicates for each treatment. Different letters mean significant differences (*p* < 0.05) **(A)**. Fungal species biodiversity: for each treatment, the frequency of isolation of the four species detected is expressed as a percentage. Isolates grown on WLN medium were grouped based on colony morphology and representatives of each colony type were identified to species level by molecular methods **(B)**. Treatment descriptions are reported in Table 1.

Three microbial groups on the grape surface were analyzed by viable count determination. Lactic Acid Bacteria (LAB, on MRS agar) were determined to be present at a concentration <10 colony forming units (CFU)/g, while Acetic Acid Bacteria (AAB, on GYC agar) were present at a concentration <100 CFU/g. The count of viable fungi is reported in Figure 2A. For all treatments, the load was between 3.5 and 4.3 Log_10_ CFU/g, corresponding to values generally reported in literature for mature grapes (Prakitchaiwattana et al., 2004). All treatments had a significantly lower fungal load compared to the CTR, which was the only treatment that had a mean load above 4 Log_10_ CFU/g. The lowest concentration (mean value 3.46 Log_10_ CFU/g) was registered for Chito and K-Bic. Aside from CTR, the highest fungal count was registered for Fos-Al (3.98 Log_10_ CFU/g) and this value was significantly higher than the loads for Chito and K-Bic.

Ten yeast isolates of each colony morphotype on WLN medium were randomly selected from different samples and subjected to PCR-RFLP of the ITS1-5.8S-ITS2 region in order to obtain an identification to species level. The species identification was confirmed for selected isolates by sequencing of the D1/D2 loop of the 26S rRNA encoding gene. The fungal identification results are shown in Figure 2B. In total, four different non-fermenting yeast species were detected on the grapes by the culture-dependent analysis. *Aureobasidium pullulans* was isolated across all treatments and in the CTR. These data are in accordance with previous studies that demonstrated *A. pullulans* is the most abundant species on grape berries at harvest time (Barata et al., 2012). Although this species is irrelevant to the winemaking industry due to an inability to tolerate ethanol, these organisms represent the resident microbiota of wine grapes (Urso et al., 2008; Alessandria et al., 2015). The presence of this microorganism on grape berries was influenced by the treatments, the frequency of isolation of *A. pullulans* ranging from around 20% for K-Pho to 100% for K-Bic, S+Met and CaO. The presence of higher populations in K-Bic, S+Met and CaO may influence the overall microbiota present on grape berries due to the antagonism of *A. pullulans* toward other yeasts and molds (Bozoudi and Tsaltas, 2018). Interestingly, an *A. pullulans*-based biopesticide has been developed and commercialized to take advantage of its ability as a biocontrol agent against pathogens (Chi et al., 2009). Consequently, the complete dominance of *A. pullulans* on grapes obtained from K-Bic, S+Met and CaO could facilitate the control of grape pathogens, as previously reported by Comitini and Ciani (2008). Interestingly, K-Bic and S+Met were two of the most effective treatments against powdery mildew (Table 1).

The second most common species was *Cryptococcus flavus*, which was identified in nine out of 11 treatments and in the CTR. This species was a minor population in all treatments except for K-Pho in which it was dominant, isolated with a frequency of almost 50% of the total colonies. *Cryptococcus terrestris* was the third species isolated in five out of 11 treatments and in CTR, but with a frequency always below 10%. *Rhodotorula graminis* was isolated only in K-Pho and in CTR: these last two treatments exhibited the highest level of biodiversity with the presence of four fungal species. Overall, these data suggest that the fungal communities that colonized “Nebbiolo” grapes were influenced by antifungal compounds. Thus, differences could be explained by the selective effect of copper and sulfur-based fungicides on the yeast ecosystem, as previously demonstrated by Grangeteau et al. (2017a) for *A. pullulans, Hanseniaspora guilliermondii, Hanseniaspora uvarum, Metschnikowia* sp., *Pichia membranifaciens, Saccharomyces cerevisiae*, and *Starmerella bacillaris* yeast isolates.

### Fungal Communities on Grapes Determined by Direct Sequencing

An appreciation of the microbial ecosystems associated with grape berry surfaces is fundamental to understanding the health status and the microbes’ contribution during alcoholic fermentation, with consequent potential impact on wine quality (Barata et al., 2012). The investigation of yeast biodiversity using culture-dependent methods can lead to incomplete microbial detection, due to low abundances or the viable but non-culturable (VBNC) yeast species (Alessandria et al., 2015). The development of next-generation sequencing (NGS) technology makes it possible to obtain more detailed information associated with wine grapes and subsequent alcoholic fermentation (Bokulich et al., 2014; Grangeteau et al., 2017b).

**FIGURE 3:**
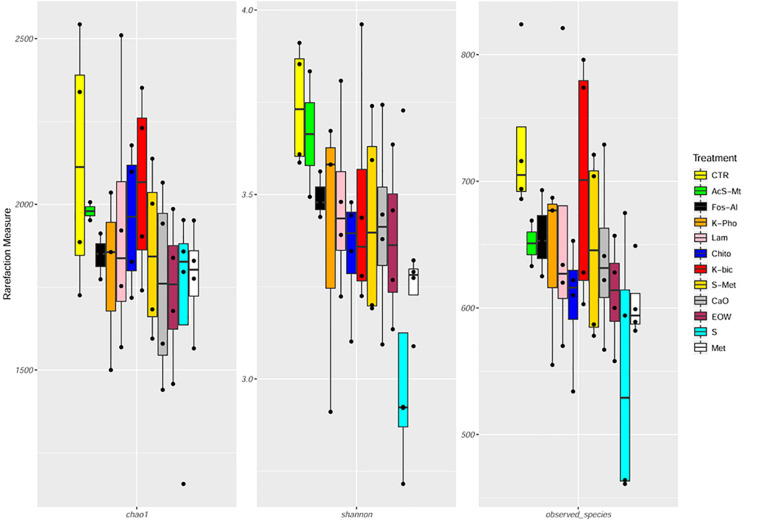
Boxplots to describe α-diversity measures of grape mycobiota. Individual points and brackets represent the richness estimate and the theoretical standard error range, respectively. Treatment descriptions are reported in Table 1.

In this study, 5 064 209 raw reads (2 × 250 bp) were obtained and 4 573 288 reads passed the filters applied through QIIME, with an average value of 103 938 ± 46 172 reads/sample, and a median sequence length of 389 bp (Supplementary Table S1). The rarefaction analysis and the estimated sample coverage indicated that there was satisfactory coverage for all the samples (ESC average 97.36%). Moreover, Shannon index (Figure 3 and Supplementary Table S1) showed a higher level of complexity (*p* < 0.05) when comparing Chito treatment to EOW as well as Chito compared to Met. No significant difference in the alpha-diversity was observed between the treatments (Figure 3 and Supplementary Table S1). Interestingly, a significant increase in the number of OTUs detected (*p* < 0.05) was observed in Chito when compared to S+Met and Met (Figure 3 and Supplementary Table S1).

Through PCA, based on the relative abundance of the OTUs (Supplementary Figure S1), a stratification of the samples according to treatment was observed. Excluding Fos-Al, which clustered together with CTR, the other treatments were well separated from the CTR (Anosim statistic *p* < 0.001). However, no clear difference between the treatments could be observed.

At harvest time 24 fungal species were identified by NGS, while only four yeast species were identified by culture-dependent methods. Regarding the most abundant OTUs shared in the entire dataset (Table 5), it was possible to observe a common mycobiota shared among treatments. The main OTUs detected were *Aureobasidium* (ranging from 24 to 38% of the relative abundance), *Cladosporium cladosporioides* (ranging from 21 to 29%), *Alternaria tenuissima* (ranging from 10 to 17%), *Pseudeurotiaceae* (ranging from 6 to 18%), *Didymella negriana* (ranging from 2 to 7%), *Saccharomyces cerevisiae* (ranging from 0.5 to 8%) and *Botrytis cinerea* (ranging from 0.2 to 13%). Regarding *S. cerevisiae*, Fleet et al. (2002) reported the low presence (populations of less than 10 CFU/g) of this species found on wine grapes using culture-dependent techniques and proposed the use of enrichment culture instead of direct agar plating. The NGS results showed *S. cerevisiae* present in all the different grapes, indicating the importance of this technique to get a better view into microbial ecosystems (Bokulich et al., 2014).

**TABLE 5:** Relative abundance of OTUs detected by 26S rRNA amplicon target sequencing.

	Treatment											
OTU	CTR	AcS-Mt	Fos-Al	K-Pho	Lam	Chito	K-Bic	S + Met	CaO	EOW	S	Met
*Alternaria*	0.878	0.851	0.998	0.730	0.823	0.785	0.798	0.753	0.672	0.702	0.552	0.724
*Alternaria tenuissima*	12.237	17.070	17.343	13.246	14.898	12.947	13.232	14.328	12.195	13.048	10.953	11.622
*Aureobasidiaceae*	0.199	0.121	0.221	0.179	0.136	0.157	0.207	0.149	0.139	0.122	0.142	0.187
*Aureobasidium pullulans*	28.221	24.499	33.897	37.853	28.532	33.529	35.532	31.996	31.759	25.447	34.057	34.444
*Botrytis cinerea*	0.086	0.082	0.213	0.586	0.089	0.085	0.118	0.166	0.154	13.519	0.060	0.124
*Cladosporium*	0.469	0.589	0.328	0.328	0.459	0.549	0.392	0.426	0.477	0.349	0.368	0.423
*Cladosporium cladosporioides*	24.870	25.839	24.817	22.090	26.665	21.633	25.030	26.343	25.752	20.908	24.867	29.187
*Cladosporium ramotenellum*	0.501	0.441	0.417	0.521	0.495	0.506	0.545	0.545	0.558	0.439	0.489	0.492
*Didymella negriana*	7.282	3.372	3.940	2.737	4.652	3.105	3.375	2.601	2.348	2.979	1.927	2.536
*Didymellaceae*	0.983	0.869	0.982	0.896	1.129	0.820	0.934	0.744	0.788	0.687	0.694	0.776
*Erysiphe necator*	14.794	15.090	8.074	13.459	15.869	20.418	12.317	15.436	17.825	7.697	18.397	13.664
*Filobasidium magnus*	1.629	1.295	1.461	1.209	0.806	0.204	0.617	1.014	1.272	0.767	0.448	0.773
*Filobasidium stepposum*	0.113	0.181	0.417	0.141	0.059	0.032	0.056	0.042	0.097	0.048	0.088	0.053
*Heterophoma novae-verbascicola*	0.136	0.112	0.070	0.103	0.148	0.145	0.116	0.098	0.124	0.091	0.078	0.094
*Lecanoromycetes*	0.448	1.579	0.664	0.511	0.480	1.126	0.779	0.596	0.740	1.322	0.874	0.810
*Melampsora apocyni*	0.155	0.078	0.080	0.213	0.075	0.124	0.189	0.186	0.103	0.048	0.152	0.261
*Microsphaeropsis*	0.201	0.435	0.300	0.370	0.456	0.275	0.376	0.284	0.255	0.192	0.248	0.332
*Nigrospora oryzae*	0.065	0.145	0.070	0.062	0.116	0.094	0.092	0.184	0.098	0.106	0.094	0.088
*Pleosporales*	1.040	1.216	0.950	0.755	1.103	0.829	0.699	0.980	0.868	0.812	0.721	0.676
*Saccharomyces cerevisiae*	0.551	3.052	0.994	0.302	0.306	0.245	1.861	0.391	0.684	8.115	0.770	0.400
*Schizosaccharomyces japonicus*	0.021	0.009	0.157	0.040	0.011	0.032	0.148	0.048	0.038	0.137	0.039	0.020
*Sporobolomyces roseus*	0.193	0.103	0.221	0.155	0.110	0.029	0.048	0.089	0.121	0.075	0.051	0.044
*Tintelnotia opuntiae*	0.737	0.181	0.384	0.292	0.189	0.359	0.134	0.181	0.198	0.130	0.465	0.193
*Vishniacozyma carnescens*	0.353	0.178	0.189	0.501	0.216	0.027	0.104	0.131	0.232	0.196	0.027	0.054
*Vishniacozyma victoriae*	0.232	0.118	0.282	0.143	0.107	0.026	0.027	0.038	0.131	0.071	0.015	0.048

*Only OTUs with an incidence above 0.2% of the relative abundance in at least two samples are shown. Abundance of OTUs in the biological replicates was averaged.*

**FIGURE 4:**
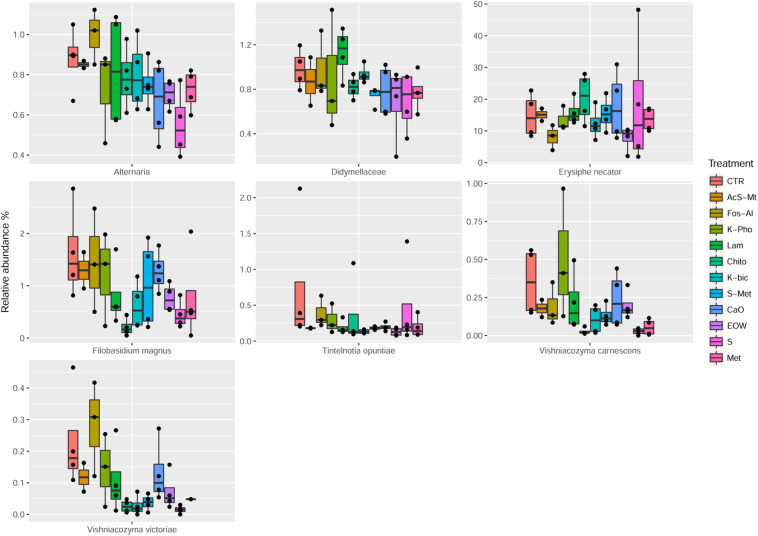
Boxplots showing the relative abundance at species or genus level of the OTUs, differentially abundant based on Wilcoxon matched pairs test, in grape samples. Treatment descriptions are reported in Table 1.

The employment of different fungicides and resistance inducers did not cause a dramatic change in the yeast genera detected by culture-independent technique. According to statistical tests, only the minor OTU fractions (ranging from 0.09 to 12%) were affected by the different treatments (Table 5). More specifically, *Alternaria* spp. were reduced by S and Met, compared to the CTR (FDR < 0.05). It should be pointed out that *Alternaria* is recognized as being among the main mycobiota populations of grape at harvest and are considered to be indicators of grape spoilage (Tournas and Katsouda, 2005). In addition, *Alternaria* is known to produce secondary metabolites (mycotoxins) that can contaminate first grapes and later the wine (Serra et al., 2006). Furthermore, *Didymellaceae* was reduced by S+Met, EOW and Met; *Heterophoma novae-verbascicola* by the use of S+Met and Met; *Vishniacozyma carnescens* and *V. victoriae* by the use of Chito, K-Bic, S and Met, while *Filobasidium magnus* was affected by Chito and S (FDR < 0.05) compared to the CTR. Nevertheless, the relevance for the microbiological quality of the grapes of the differences described above is not expected to be important due to the low abundance of these populations. Interestingly, it was possible to detect by a culture-independent approach *E. necator* in grape berries of the CTR and all the treatments applied. It should be noted, that *E. necator* was the only population that could be considered of relevance due to the high relative abundance obtained in all the samples (range 8–20%, average 15%). Regarding the effect of the various treatments on the population of *E. necator*, we observed a reduction when EOW was used only if compared with Chito and Lam (Figure 4).

The mycobiota composition was also tested for possible correlations with the disease index and berry skin mechanical properties obtained for each of the treatments as well as the CTR. As shown in Supplementary Figure S2, we observed a positive correlation between *Cladosporium cladosporioides* and berry skin break force (FDR < 0.05). *C. cladosporioides* was the second most abundant population in samples from all treatments and the CTR. However, no significant differences were observed in the relative abundance between the various treatments. This result implies that this species was not influenced by the treatment applied in the vineyard. The presence of *Cladosporium* genus, in particular *Cladosporium herbarum* and *Cladosporium cladosporioides*, on grapes is associated with the development of *Cladosporium* rot (Barata et al., 2012). Recently, it has been shown that the population of *Cladosporium* increases as grapes mature and it may develop on the surface of apparently healthy grapes (Briceño and Latorre, 2008). The positive correlation observed, i.e., increased grape skin force associated with the presence of *C. cladosporioides*, would require further investigation in order to confirm this correlation for other grape varieties and in subsequent harvests, and more importantly to establish the cause-effect relationship of the observations made in this study. Also the presence of *Heterophoma novae-verbascicola* was correlated with berry skin break force (FDR < 0.05). Furthermore, a direct association of % cluster and berries affected by powdery mildew with *Filobasidium stepposum*, *Vishniacozyma victoriae* and *Vishniacozyma carnescens* was observed. In the case of these last correlations revealed by statistical analysis, the OTUs involved were present at a very low abundance (<1%), are considered minor and therefore it would be important to confirm this association using RNA as a target as it is a better indicator of vitality compared to DNA, and if possible to obtain quantitative data by performing viable counts.

### Wine Chemical-Physical Characteristics

The effects on wine of K-Bic (the most effective biocontrol product in reducing powdery mildew) were evaluated in comparison with wine produced from CTR and the standard control strategy (S+Met). Significant differences (*p* < 0.05) were found among treatments for almost all parameters tested (Supplementary Table S2). The ethanol content well reflected the average values found for soluble solids on the grape must, while the differences in acidity parameters were not always in line with grape berry measurements. All wines performed the malo-lactic fermentation, leading to no residual malic acid detected in the wines, and the tartaric acid stabilization. Among acidity values, it is worth noting the acetic acid trend, highlighting reduced contents for K-Bic and particularly for S+Met treatment, resulting in significant differences (*p* < 0.001) for all wines produced (Supplementary Table S2).

Despite the reduced ethanol content, the S+Met trial achieved the highest quantities of anthocyanins and total phenols (*p* < 0.01), but these differences were not reflected in the flavanols content, analyzed by both high-mass tannins (proanthocyanidins assay; *p* > 0.05) and low-mass compounds (vanillin assay; *p* > 0.05). The differences in the anthocyanin richness, the principal compounds responsible for the red color of young wines, had a decisive impact on the color intensity and on the CIEL^∗^a^∗^b^∗^ a^∗^ coordinate (*p* < 0.01). Both K-Bic and S+Met treatments resulted in a wine color with a ΔE^∗^ above 3 when compared to CTR, a threshold indicating the potential ability of the human eye to recognize the color differences during a tasting (Martínez et al., 2001).

## Conclusion

This study was focused on possible indirect effects of vineyard treatments with chemical, biocontrol products and resistance inducers against downy and powdery mildews on “Nebbiolo” grapes at harvest. Treatment with potassium bicarbonate (K-Bic) was the most effective in reducing powdery mildew on grape clusters with an efficiency similar to the standard control strategies S+Met and S applied, respectively, in conventional and organic farms.

The yield and vigor of vines, as well as the primary and secondary metabolites produced in the berries were not influenced by the different treatments. On the contrary, the active ingredients present in the treatments modified the mechanical properties of the skin (hardness and thickness). In particular, a slight hardening of the skin was detected when laminarin (Lam) and chito-oligosaccharides and oligogalacturonides (Chito) were used, while treatments with sulfur (S and S+Met) and K-Bic induced a thickening of the skin. Interestingly, these treatments were the most effective treatment against powdery mildew. In addition to mechanical properties, the total yeast community present on grape berries was also modified in response to antifungal treatments. *A. pullulans* was the dominant species and its relative abundance was influenced by the compounds used in this study. In particular, *A. pullulans* is also known as biocontrol agent against pathogens and a higher population was detected in berries treated with K-Bic and S+Met, products particularly effective against powdery mildew. *Alternaria* was reduced by treatments and this can positively affect the quality and the safety of the grapes. The effect of such treatments on population kinetics during spontaneous or inoculated alcoholic fermentation deserves further investigation.

## Data Availability Statement

The datasets generated for this study can be found in the Sequence Read Archive of NCBI, BioProjectID PRJNA575160.

## Author Contributions

KR, SG, and MP performed most of the experiments, analyzed and compared the data. KR, VE, and IF performed the microbiological analysis and bioinformatic elaboration of data. SG and SR carried out the chemical and texture analyses. MP and MM performed the pathological analyses. IG carried out the agronomical measurements, analyzed the corresponding data. MG supported the pathological analyses and critically revised the manuscript. GG, LR, MP, and KR conceived the project, supervised all the experiments and wrote the manuscript. All authors read and approved the manuscript.

## Conflict of Interest

MM was employed by company ANT-NET srl. The remaining authors declare that the research was conducted in the absence of any commercial or financial relationships that could be construed as a potential conflict of interest.

## Footnotes

1 http://www.ncbi.nlm.nih.gov/blast/

2 www.r-project.org
