# Supplementary material for: Impact of Chemical and Alternative Fungicides Applied to Grapevine cv Nebbiolo on Microbial Ecology and Chemical-Physical Grape Characteristics at Harvest
Source: Front Plant Sci. 2020 May 29;11:700. doi: 10.3389/fpls.2020.00700 (PMC7272676; doi:10.3389/fpls.2020.00700)
Supplement: Supplementary file 1 [file Data_Sheet_1.PDF]

Impact of chemical and alternative fungicides applied to grapevine cv Nebbiolo on microbial ecology and chemical-physical grape characteristics at harvest

Kalliopi Rantsiou, Simone Giacosa, Massimo Pugliese, Vasileios Englezos, Ilario Ferrocino, Susana Río Segade, Matteo Monchiero, Ivana Gribaudo, Giorgio Gambino, Maria Lodovica Gullino, Luca Rolle

Supplementary Figures

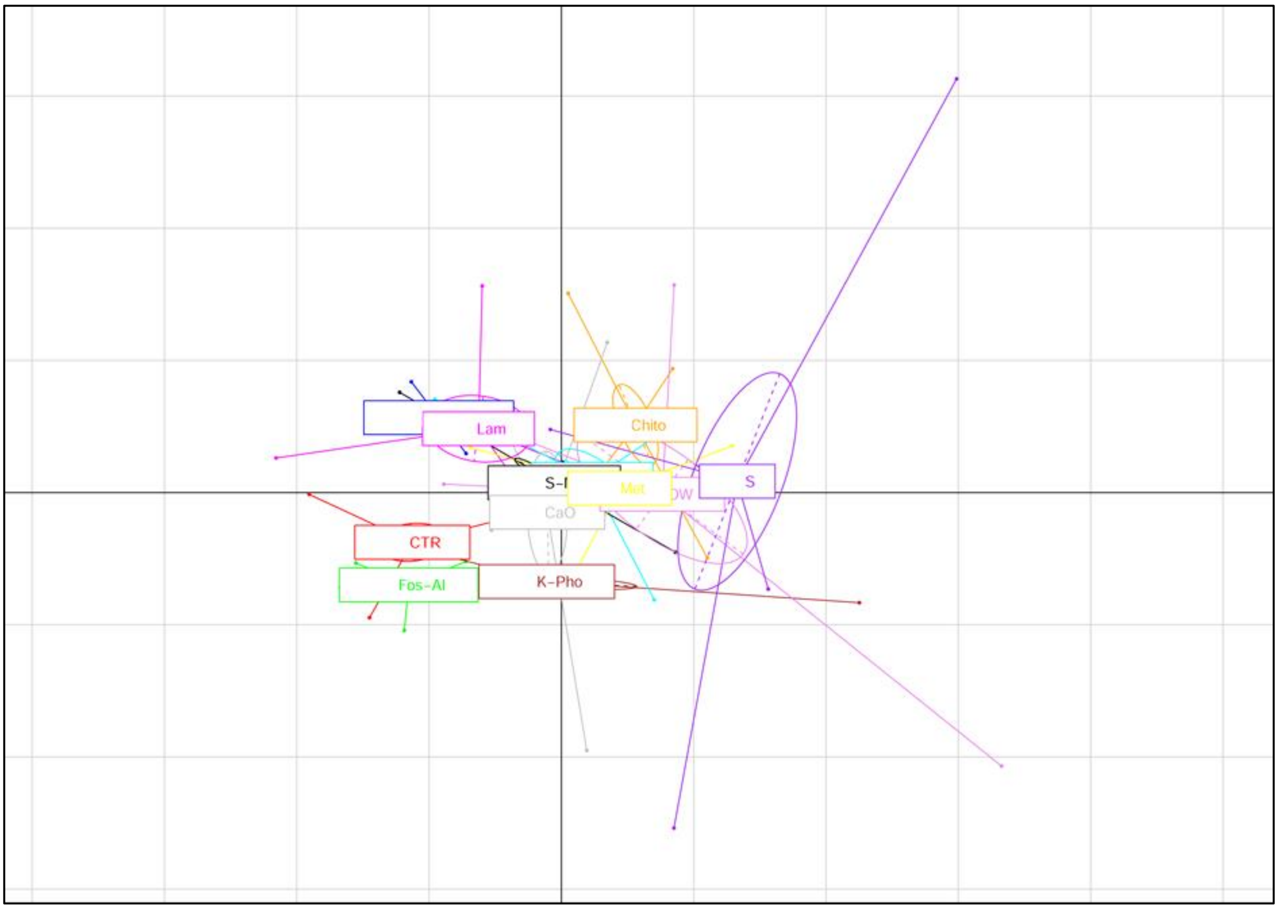

Figure S1. Principal Component Analysis (PCA) based on OTUs' relative abundance in the grape mycobiota. Samples are colour coded according to treatment. The first component (horizontal) accounts for 25.11% of the variance and the second component (vertical) accounts for 20.55%. Treatment descriptions are reported in Table 1.

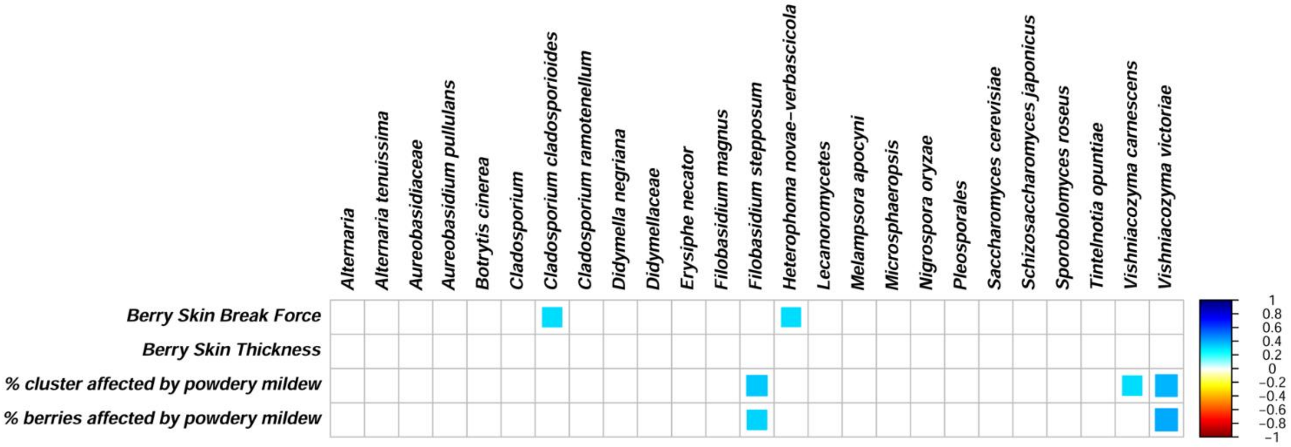

Figure S2. Spearman's rank correlation matrix of mycobiota abundance, disease index and berry skin mechanical properties. The colours of the scale bar denote the nature of the correlation, with 1 indicating a positive correlation (blue) and -1 indicating a negative correlation (dark red) between the two datasets. Only correlations with an FDR value lower than 0.05 are displayed.
